# Supplementary material for: Human Intramuscular Hyperimmune Gamma Globulin (hIHGG) Anti-SARS-CoV-2—Characteristics of Intermediates and Final Product
Source: Viruses. 2022 Jun 17;14(6):1328. doi: 10.3390/v14061328 (PMC9227433; doi:10.3390/v14061328)
Supplement: Supplementary file 1 [file viruses-14-01328-s001.zip › viruses-1737365-supplementary.pdf]

Supplemental materials

Material and methods

Neutralization assay for the presence of antibodies with SARS-CoV-2 on the Vero cell line (VN)

Virus neutralization (VN) assay was used with SARS-CoV-2 (Munchen-1.2 2020/984) on the Vero cell line. The preparations were diluted in culture medium and mixed with SARS-CoV-2 virus. Positive control were virus infected cells in the absence of the tested substance (globulin dilutions). Negative control were mock infected cells. The final titer of 1600 TCID50 / ml was used. After 72 hours of incubation the cells were analyzed for the cytopathic effect.

Neutralization assay with pseudovirus (PVN)

Pseudovirus generation

Briefly, 293T cells were seeded on 10 cm^2 cell dishes, cultured for 24 h at 37°C with 5% CO_2, and transfected using polyethyleneimine (Sigma-Aldrich, Poland) with the lentiviral packaging plasmid (psPAX), VSV-G envelope plasmid (pMD2.G) or SARS-CoV-2 S glycoprotein (pCAGGS-SARS-CoV-2-S) and third plasmid encoding luciferase protein (pRRL Luciferase plasmid was a gift from Paul Khavari (Addgene plasmid #120798; http://n2t.net/addgene:120798; RRID: Addgene_120798). Cells were further cultured for 72 h at 37°C with 5% CO_2. Medium was changed after 24 h, and pseudoviruses were collected after 48 and 72 h. Next, collected pseudoviruses were filtered by 0.22 µm filter, 10-times concentrated with Amicon Ultra-15 Centrifugal Filters (10k, Merck Millipore), and stored at -80 °C.

Neutralization assay with pseudoviruses

For detailed determination of sera's neutralization properties, sera obtained from patients were heat-inactivated in 56°C for 30 min. Two-fold sera dilutions were then preincubated with pseudoviruses harboring S-SARS-CoV-2 in a volume ratio of 1:1 for 1h at RT. Subsequently, samples were applied to the $A549^{ACE2+}$ cells at 30-50% confluency, seeded in culture medium on 96-well plates 2 days before transduction. Control cells were transduced with pseudoviruses harboring VSV-G protein and pseudoviruses lacking envelope protein (ΔEnv) as positive and negative control, respectively. Transduction with pseudoviruses was carried out in the presence of polybrene (4 µg/ml; Sigma-Aldrich, Poland). After 4 h of incubation at 37°C, cells were rinsed twice with PBS. Fresh medium was added. The neutralization assay was carried out for 72 h. Culture supernatants were removed by washing twice with PBS. For chemiluminescence detection 100 µl of BrightGlo reagent (Promega) was added and incubated for 5 min to lyse the cells and the supernatant was transferred to the measuring plate. Luminescence was measured as the final step.

Table S1. Blood group of donors whose plasma was used for production of hIHGG (0102).

**Blood group**	**Donation number**	**Percentage** (%)
0-	7	3.61
0+	51	26.29
A-	14	7.22
A+	77	39.69
AB-	4	2.06
AB+	12	6.19
B-	9	4.64
B+	20	10.31

Tabela 2. Reactivity of immunoenzymatic tests anti-S1, anti-RBD S1 and anti-N as well as neutralizing activity in the assays of successive intermediates obtained in the production of hyperimmune gamma globulin anti-SARS-CoV-2 (batch 0120).

Results for anti-S1 RBD and anti-N antibodies were expressed as S/Co (absorbance value divided by the cut-off value) and anti-S1 IgG in the semi-quantitative EIA (Euroimmun) S/Co (Ratio) test and quantitative results in RU/ml (relative units/milliliter). The Euroimmun test was calibrated against the first international WHO anti-SARS-CoV-2 standard, so the results were expressed in international units - BAU/ml (binding antibody units/milliliter).

a. plasma pool

	**Dilution**	**anti-S1**			**anti-RBD S1**		**anti-N** (S/Co)	**Neutralization**	
		IgG quant In RU/ml (BAU/ml)	IgG (S/Co)	IgA (S/Co)	Total (S/Co)	IgM (S/Co)		VN	PVN [%]
**Control samples**	Donation Z5320 xxxxxxxx				0.04	0.1	0.011		
	Gamma anti-D 150 s.0120/0420		0.237		0.06	0.1	0.4		
	Gamma anti-D 50 s.0220		0.217		0.05	0.1	0.2		
**Dilution**	No dilution	**71.61** (229.151)	**3.82**	**3.37**	**> 19.9**	**4.1**	**> 19.9**	nt	nt
	1:20	2.86	0.27	nt	**5.838**	**3.7**	0.642	**+++**	nt
	1:40	1.09	0.17	nt	**2.873**	**2.7**	0.432	**+++**	**75.40**
	1:80	0.2	0.09	nt	**1.468**	**1.5**	0.243	**++**	36.44
	1:160	0	0.07	nt	0.642	0.8	0.276	**+**	27.84
	1:320	0	0.04	nt	0.318	0.4	0.291	**+**	0
	1:640	0	0.04	nt	0.156	0.2	0.304	**+**	4.12
	1:1280	0	0.03	nt	0.092	0.6	0.302	**+**	14.01
	1:2560	nt	0.04	nt	0.069	0.5	0.143	-	0
	1:5120	nt	nt	nt	0.069	0.5	0.291	-	3.28
**Titer**(estimated)		**7.807**	**4.213**		**110.897**	**115.679**	**13.628**		**IC_{50} 63.706**

b. Concentrate after fractionation, before lyophilization

	Dilution	anti-S1			anti-RBD S1		anti-N	Neutralization
		IgG quant<br>In RU/ml (BAU/ml)	IgG<br>(S/Co)	IgA<br>(S/Co)	Total<br>(S/Co)	IgM<br>(S/Co)	(S/Co)	VN
**Control samples**	Donation Z5320 xxxxxxxx				0.04	0.1	0.011	
	Gamma anti-D 150 s.0120/0420		0.237		0.06	0.1	0.4	
	Gamma anti-D 50 s.0220		0.217		0.05	0.1	0.2	
**Dilution**	No dilution	**>120**	**9.12**	0.41	**> 19.9**	**3.4**	**> 19.9**	nt
	1:20	**17.33** (55.456)	**1.81**	0	**> 19.9**	**1**	**3.885**	**+++**
	1:40	10.83	0.96	nt	**15.272**	0.4	**2.1**	**+++**
	1:80	5.57	0.41	nt	**8.035**	0.2	0.98	**+++**
	1:160	2.42	0.24	nt	**3.676**	0.1	0.622	**+++**
	1:320	0.6	0.14	nt	**1.555**	0.1	0.452	**++**
	1:640	0	0.08	nt	0.809	0.1	0.365	**++**
	1:1.280	1.4	0.05	nt	0.289	0.1	0.221	**+/-**
	1:2.560	nt	0.04	nt	0.127	0.1	0.294	-
	1:5.120	nt	nt	nt	0.081	0.1	0.317	-
**Titer (estimated)**			**33.343**	**0**	**470.784**	**20**	**84.045**	

c. 15% concentrate

	Dilution	anti-S1			anti-RBD S1		anti-N (S/Co)	Neutralization	
		IgG quant In RU/ml (BAU/ml)	IgG (S/Co)	IgA (S/Co)	Total (S/Co)	IgM (S/Co)		VN	PVN [%]
**Control samples**	Donation Z5320 xxxxxxxx				0.04	0.1	0.011		
	Gamma anti-D 150 s.0120/0420		0.237		0.06	0.1	0.4		
	Gamma anti-D 50 s.0220		0.217		0.05	0.1	0.2		
**Dilution**	No dilution	**>120**	**8.86**	0.57	**> 19.9**	**2.9**	**> 19.9**	nt	nt
	1:20	**29.28** (93.696)	**2.98**	0.04	**> 19.9**	**1.4**	**> 19.9**	**+++**	nt
	1:40	**16.37** (52.384)	**1.66**	nt	**>19.9**	0.7	**4.157**	**+++**	**101.23**
	1:80	11	0.88	nt	**11.942**	0.4	**2**	**+++**	**92.92**
	1:160	5.48	0.47	nt	**8.96**	0.2	**1.081**	**+++**	**81.68**
	1:320	2.37	0.25	nt	**3.682**	0.1	0.726	**+++**	**73.45**
	1:640	0.56	0.12	nt	**1.919**	0.1	0.456	**+++**	**64.63**
	1:1.280	0	0.07	nt	0.890	0.1	0.360	**+++**	47.46
	1:2.560	nt	0.06	nt	0.434	0.1	0.360	**++**	39.77
	1:5.120	nt	nt	nt	0.0202	0.1	0.291	**++**	28.17
	**Titer (estimated)**		**62.750**	**0**	**1264**	**29.334**	**175.802**		IC_{50} **1278.151**

d. 10% concentrate

	Dilution	anti-S1			anti-RBD S1		anti-N (S/Co)	Neutralization	
		IgG quant In RU/ml (BAU/ml)	IgG (S/Co)	IgA (S/Co)	Total (S/Co)	IgM (S/Co)		VN	PVN [%]
**Control samples**	donation Z5320 xxxxxxxx				0.04	0.1	0.011		
	Gamma anti-D 150 s.0120/0420		0.237		0.06	0.1	0.4		
	Gamma anti-D 50 s.0220		0.217		0.05	0.1	0.2		
**Dilution**	No dilution	**>120**	**8.56**	0.28	**>19.9**	**3.1**	**>19.9**	nt	nt
	1:20	**29.28** (93.696)	**2.31**	0.02	**>19.9**	**1.07**	**5.2**	**+++**	nt
	1:40	**16.37** (52.384)	**1.23**	nt	**18.543**	0.5	**2.5**	**+++**	**95.76**
	1:80	11	0.51	nt	**10.231**	0.2	**1.395**	**+++**	**83.34**
	1:160	5.48	0.34	nt	**5.642**	0.1	0.756	**+++**	**61.04**
	1:320	2.37	0.18	nt	**2.416**	0.1	0.551	**+++**	**55.04**
	1:640	0.56	nt	nt	**1.214**	0.1	0.393	**+++**	48.40
	1:1.280	0	0.30	nt	0.566	0.1	0.321	**+++**	28.35
	1:2.560	nt	0.06	nt	0.277	0.1	0.341	**++/+**	22.28
	1:5.120	nt	nt	nt	0.116	0.1	0.211	**++/-**	19.66
	**Titer (estimated)**		**42.567**	**0**	**781.132**	**21.415**	**108.554**		IC$_{50}$ **402**

e. after ampoule filling

	Dilution	anti-S1 IgG (S/Co)	anti-S1 IgA (S/Co)	anti-RBD S1 Total (S/Co)	anti-RBD S1 IgM (S/Co)	anti-N (S/Co)	neutralization VN	neutralization PVN [%]
**Control samples**	Donation Z5320 xxxxxxxx			0.04	0.1	0.147		
	Gamma anti-D 150 s.0120/0420	0.237		0.06	0.1	0.4		
	Gamma anti -D 50 s.0220	0.217		0.05	0.1	0.2		
**Dilution**	No dilution	**12.47**	0.39	**>19.9**	**2.8**	**>19.9**	nt	nt
	1:20	**2.83**	0	**>19.9**	**1.2**	**>19.9**	**+++**	nt
	1:40	**1.69**	0	**>19.9**	0.6	**2.105**	**+++**	**96.89**
	1:80	0.79	0	**11.9**	0.3	**1.046**	**+++**	**88.63**
	1:160	0.7	0	**5.9**	0.1	0.631	**+++**	**71.03**
	1:320	nt	nt	**2.77**	0.1	0.445	**++**	47.54
	1:640	nt	nt	**1.34**	0.1	0.299	**++**	38.05
	1:1.280	nt	nt	0.57	0.1	0.260	**+**	32.15
	1:2.560	nt	nt	0.2	0.1	0.203	**+**	27.16
	1:5.120	nt	nt	0.1	0.1	nt	-	3.69
	1:10.240	nt	nt	nt	nt	nt	-	nt
**Titer (estimated)**		**63.309**	**0**	**801.840**	**24.439**	**87.577**		IC_{50} **313.549**
